# Herpesvirus Vaccines

**DOI:** 10.3390/vaccines10040628

**Published:** 2022-04-18

**Authors:** Stefano Petrini, Peter Maple

**Affiliations:** 1 National Reference Centre for Infectious Bovine Rhinotracheitis (IBR), Istituto Zooprofilattico Sperimentale Umbria-Marche, Via Gaetano Salvemini 1, 06126 Perugia, Italy; 2 Division of Clinical Neuroscience, Section of Clinical Neurology, University of Nottingham, Nottingham NG7 2UH, UK; eastyorksmicrobiol@gmail.com; 3 Department of Neurology, Nottingham University Hospitals NHS Trust, Nottingham NG7 2UH, UK

The Special Issue titled “Herpesvirus Vaccines” contains different articles and a review regarding veterinary and human herpesviruses. The Special Issue covers topics including *Bovine alphaherpesvirus 1* (BoHV-1), *Infectious laryngotracheitis virus* (ILTV), *Equine alphaherpesvirus 1* (EHV-1), *Equine alphaherpesvirus 4* (EHV-4), *Human Cytomegalovirus virus* (HCMV), and *Epstein–Barr virus* (EBV).

The open manuscript by Petrini et al. [1] suggests the urgency of developing strategies to eradicate *Bovine alphaherpesvirus 1* (BoHV-1) in both cattle and water buffalo species. To date, in Europe, glycoprotein E (gE)-deleted marker vaccines against BoHV-1 are commercially available only for the cattle industry. For the first time, the manuscript evaluates the safety and efficacy of a commercial inactivated gE-deleted marker vaccine (Bovilis ^®®^ IBR marker inactivatum, Intervet International B.V. Boxmeer, Holland) in water buffalo. Ten water buffaloes were divided into two groups (A = vaccinated; B = controls) with five animals each. The only animals in group A were injected via intramuscular route. Sixty days after the first immunization, all animals were challenged with a wild-type BoHV-1 strain via the intranasal route. The BoHV-1 immune response was detected in group A 30 days post-vaccination, whereas the antibodies appeared 10 days post-challenge in group B. Moreover, group A did not demonstrate viral shedding or clinical signs, unlike group B. However, post-challenge, the BoHV-1 humoral and cell-mediated immune responses increased more dramatically in group A compared to group B. In conclusion, the results of this study indicate that the vaccination of *Bubalus Bubalis*, with the above-mentioned product, was able to protect the water buffaloes against wild-type BoHV-1 strain. Finally, the buffalo’s marker vaccine can be used under the new European Regulations, so-called “Animal Health Law”, which can control BoHV-1 in cattle and *Bubalus Bubalis*.

In a double-blind, randomized clinical trial, Attili et al. [2] investigate three different vaccination protocols against *Equine alphaherpesvirus 1* (EHV-1) and *Equine alphaherpesvirus 4* (EHV-4) infection in mares. All the animals were regularly vaccinated during the past years with the same vaccine (Duvaxyn EHV1/4, Fort Dodge, in the past, and Equip EHV 1, 4, Zoetis, nowadays). Eighteen mares were selected and divided into three groups (G1-G2-G3). A further group was added as a negative control (Ctrl). G1 received the vaccine at the third, fifth, and seventh months of pregnancy; G2 at the fifth, seventh, and ninth months of pregnancy, as suggested by the manufacturer; and G3 7 days before the expected date of birth and at the first, fourth, and sixth months of pregnancy. The vaccine was administered via intramuscular route. Nasal swabs, blood, and serum samples were collected for virological (PCR investigations) and serological investigations (ELISA and seroneutralization tests) at different experimental times. The results evidenced that the protocol used in G3 (4 doses) increased the titer recorded by ELISA and seroneutralization (SN) tests. There was no correlation in titer values between ELISA and SN and between SN and PCR. A very weak positive correlation between ELISA and PCR was obtained. Virological positivity was evidenced from 7 out of 18 nasal swabs. Viremia and abortion were not observed. Furthermore, the study was conducted in field conditions in a population with a known history of infection and abortion. In conclusion, the protocol proposed in group G1 is the least efficient, while the protocol evaluated in G3 seems to have induced a higher antibody titer in both SN and ELISA. However, in this study, the high concentration of antibodies during the first vaccination significantly affected the immune response by inducing a rapid decline in antibodies. Furthermore, no data were obtained on the protection against abortion as there was no abortion control group. For these reasons, further studies are required to assess whether vaccination in mares during pregnancy and the type of protocol should be considered to reduce the occurrence of abortion.

To date, marker vaccines are commonly used in Europe, but their ability to induce passive immunity is poorly known. The manuscript by Petrini et al. [3] evaluates the passive immunity transferred from dams immunized with commercial inactivated gE-deleted marker vaccine to calves. The authors immunized 12 pregnant cattle devoid of neutralizing antibodies against *Bovine alphaherpesvirus 1* (BoHV-1) and divided them into two groups with six animals each. Both groups were injected with a different inactivated gE-deleted marker vaccine administrated via the intranasal or intramuscular route. An additional group of six pregnant cattle served as the unvaccinated control group. After calving, the number of animals in each group was increased by the newborn calves. In the dams, the humoral immune response was evaluated before calving and, subsequently, at different times until post-calving day 180 (PCD 180). In addition, the antibodies in colostrum, milk, and serum samples from newborn calves were evaluated at different times until PCD 180. The results indicate that the two inactivated glycoprotein E (gE)-deleted marker vaccines against BoHV-1 are safe for pregnant cattle and effectively transferred passive immunity from dams to calves up to PCD 180. In conclusion, the products tested in this study are safe and suitable for immunization in BoHV-1 eradication programs. Finally, the authors report that further research is needed to assess whether passive immunity in calves can protect them against experimental infection using a BoHV-1 virulent virus.

An article by Ozan Atasoy et al. [4] developed and applied a highly efficient, versatile, and rapid NHEJ-CRISPR/Cas9 and Cre-Lox-mediated genome editing approach for the simultaneous deletion of pre-determined virulence factors and insertion of viral antigen to generate recombinant, multivalent, and safer vaccine vectors. The authors demonstrated the use of this approach, first by generating a report virus and by deleting the TK gene from infectious laryngotracheitis virus (ILTV). Later, the authors engineered an ILTV-vector vaccine candidate by harboring fusion gene (F) of the velogenic NDV and deleting another gene, US4, from the ILTV genome. The reporter marker gene was excised using the Cre–Lox system without affecting the expression and stability of the F protein. This potential vaccine candidate was evaluated for the stable expression of the inserted protein, replication kinetics, and comparative in vitro characteristics. The authors demonstrate that NHEJ-CRISPR/Cas9 accompanied by the Cre–Lox platform is an efficient method for the rapid generation of ILTV-based recombinant vaccines and propose the multiple advantages of traditional recombinant and recombineering techniques.

To date, nine human herpesviruses have been classified [5] and all can cause severe disease, particularly when the immune system is weakened, deficient, or under-developed. Herpesvirus infections or reactivations are a major problem in transplant patients [6], as well as in people infected with HIV [7], and infection during pregnancy may result in fetal developmental abnormalities [8]. Certain herpesviruses are oncogenic [9] and herpesvirus infections may contribute to the development of autoimmune-mediated diseases, such as multiple sclerosis [10]. For all these reasons, the development of human herpesvirus vaccines can be beneficial; however, only vaccines against chickenpox (varicella-zoster virus infection) and shingles (varicella-zoster virus reactivation) are currently in clinical use. The development of human cytomegalovirus (CMV) vaccines is a high priority and several vaccine candidates produced using different technological approaches have been evaluated [11].

In this Special Issue, Cui and colleagues [12] present new evidence that the immunization of rabbits with mixtures of CMV envelope proteins can produce synergistic neutralizing antibody responses. These data support the combination of CMV core fusion machinery envelope proteins gB + gH/gL or the combination of gB and pentameric complex as ideal vaccine candidates for inducing optimal immune responses against CMV infection. A final paper [13] reviews the development of vaccines for congenital cytomegalovirus infection and briefly assesses the case for Epstein–Barr virus prophylactic vaccination for multiple sclerosis.
